# Who fans the flames of Alzheimer's disease brains? Misfolded tau on the crossroad of neurodegenerative and inflammatory pathways

**DOI:** 10.1186/1742-2094-9-47

**Published:** 2012-03-07

**Authors:** Norbert Zilka, Zuzana Kazmerova, Santosh Jadhav, Peter Neradil, Aladar Madari, Dominika Obetkova, Ondrej Bugos, Michal Novak

**Affiliations:** 1 Institute of Neuroimmunology, 84510 Bratislava, Slovak Republic; 2 Axon Neuroscience GmbH, 1030 Vienna, Austria; 3 Small Animals Clinic, University of Veterinary Medicine and Pharmacy, Komenskeho 73, 041 81 Kosice, Slovakia

**Keywords:** Alzheimer's disease, Tauopathies, Neurofibrillary degeneration, Neuroinflammation, Microglia

## Abstract

Neurodegeneration, induced by misfolded tau protein, and neuroinflammation, driven by glial cells, represent the salient features of Alzheimer's disease (AD) and related human tauopathies. While tau neurodegeneration significantly correlates with disease progression, brain inflammation seems to be an important factor in regulating the resistance or susceptibility to AD neurodegeneration. Previously, it has been shown that there is a reciprocal relationship between the local inflammatory response and neurofibrillary lesions. Numerous independent studies have reported that inflammatory responses may contribute to the development of tau pathology and thus accelerate the course of disease. It has been shown that various cytokines can significantly affect the functional and structural properties of intracellular tau. Notwithstanding, anti-inflammatory approaches have not unequivocally demonstrated that inhibition of the brain immune response can lead to reduction of neurofibrillary lesions. On the other hand, our recent data show that misfolded tau could represent a trigger for microglial activation, suggesting the dual role of misfolded tau in the Alzheimer's disease inflammatory cascade. On the basis of current knowledge, we can conclude that misfolded tau is located at the crossroad of the neurodegenerative and neuroinflammatory pathways. Thus disease-modified tau represents an important target for potential therapeutic strategies for patients with Alzheimer's disease.

## Neurodegenerative niche in the ocean of the brain inflammation

Alzheimer's disease (AD), the major cause of dementia, is characterized by the aberrant folding of the protein tau, leading to its intracellular and extracellular accumulation and to β-amyloidosis seen as extracellular deposits of β-amyloid (Aβ) in the brain parenchyma and around cerebral blood vessels [1-8]. Although it is well-documented that Aβ deposition is considered to be an important inducer of the chronic inflammatory response driven by activated microglia and astrocytes [9-12], little is known about the role of misfolded tau in the neuroinflammatory cascade. In AD, the pathological lesions of misfolded tau are present as intracellular and extracellular neurofibrillary tangles, neuropil threads and neuritic plaques [6,7]. Interestingly, several independent studies have revealed that the regional distribution and load of neurofibrillary lesions parallel the distribution of reactive microglia in AD [13,14]. In an extensive histopathological study published by Irina Alafuzoff's group, it was shown that ApoE genotype significantly influenced the linkage between neurofibrillary tangles (NFTs) and activated microglia. Furthermore, the authors clearly demonstrated that microglial upregulation of major histocompatibility complex class II antigen (HLA-DR) increased the duration of AD and correlated with NFT counts in sporadic cases, but not in familial ones [15].

**Figure 1:**
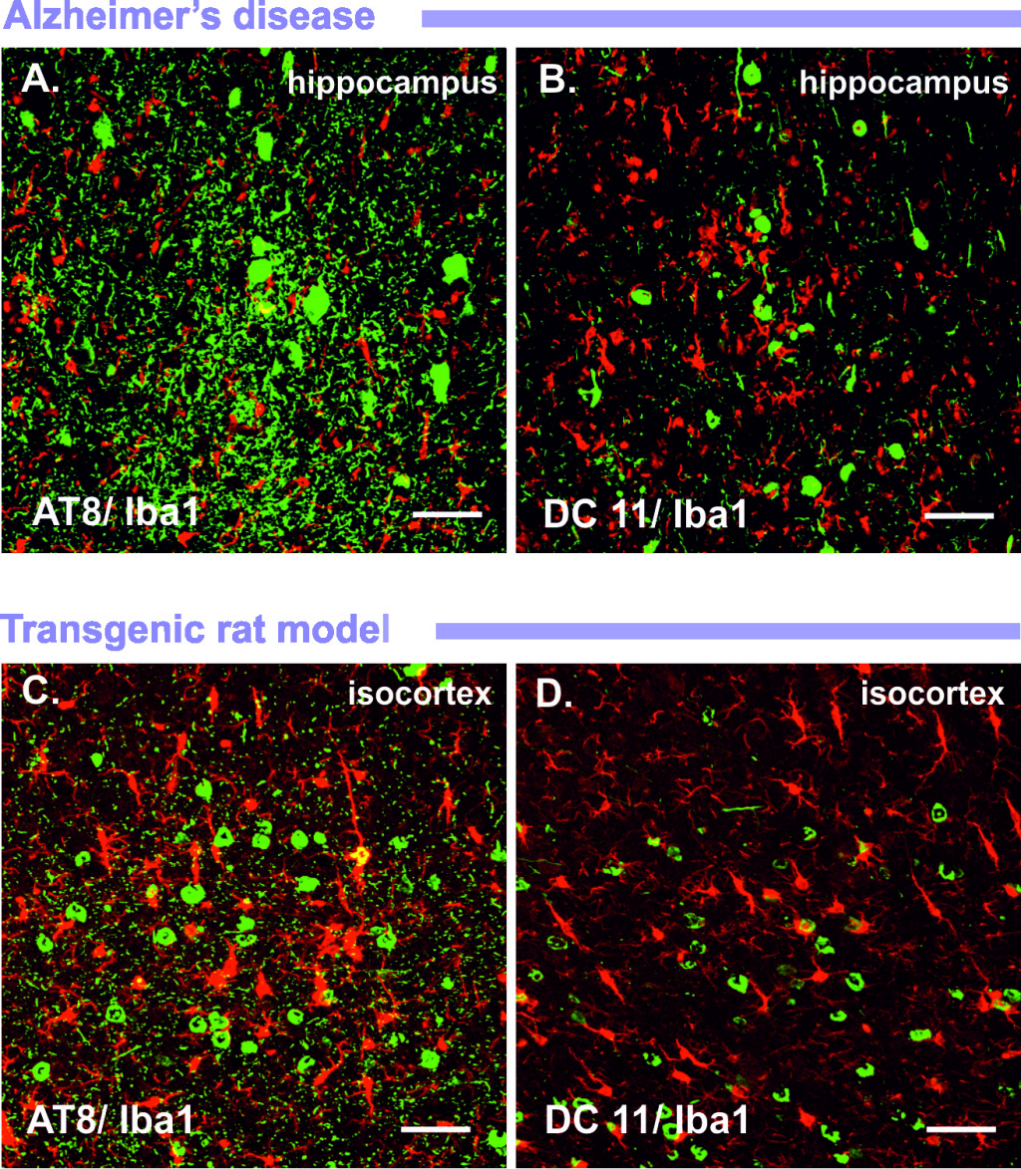
**Activated microglia are localized in brain areas affected by neurofibrillary tangles**. Reactive microglia, stained by Iba1 antibody (red colour), are distributed throughout the brain regions affected by neurofibrillary lesions immunolabelled with AT8 (antibody recognizing phospho-tau, green colour) and DC11 (antibody recognizing Alzheimer's disease (AD)-specific misfolded tau, green colour). Activated microglia are surrounding tangle-bearing neurons in the hippocampus of an AD patient **(A) **and **(B**) and in the cortices of transgenic rats expressing truncated tau protein **(C) **and **(D)**. Scale bars: 100 μm.

Apparently, activated microglia were frequently present in the proximity of NFTs at early [16] and later stages of tangle formation [14,17,18] (Figures 1A and 1B). Sheng *et al. *showed that the number of IL-1α-positive microglia and S100β-positive astrocytes progressively increased with NFT load, suggesting that both executive arms of the brain immune system, microglia and astroglia, are involved in the immune response targeting tau pathology [16]. It is noteworthy that, in the late stages of tangle development, astrocytes and microglia infiltrate extracellular "ghost" tangles and may contribute to the degradation of tau filaments [17,19]. Likewise, the complement proteins C1q, C3d and C4d were found to colocalize with dystrophic neurites, neuropil threads and tangle-bearing neurons. Similarly, an antibody recognizing the C5b-9 membrane attack complex stained dystrophic neurites and many tangle-bearing neurons [20,21].

**Figure 2:**
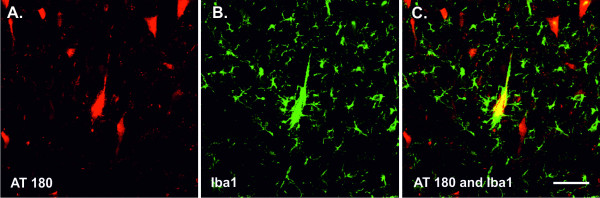
**Colocalization of clustered microglia and neurofibrillary tangles in transgenic rat brain**. Neurofibrillary tangles (NFTs) were detected with the monoclonal antibody AT180, recognizing tau protein phosphorylated at Thr231 and Ser235 **(A)**, respectively. Activated microglia were stained with the polyclonal antibody Iba1, specific for ionized calcium-binding adaptor molecule 1 **(B)**. Confocal study showed that some NFTs colocalized with clusters of activated microglia **(C)**. Scale bars: 100 μm.

It has been clearly demonstrated that microglial activation also correlates with tau lesions in other human tauopathies, such as Guam parkinsonism dementia, Pick's disease, progressive supranuclear palsy and corticobasal degeneration [22-27]. The activation of microglia linked to tau deposition has been well-documented in various transgenic rodent models expressing human mutant tau protein P301S [28,29], R406W [30], P301L [31] or disease-modified, truncated tau protein [32,33]. We have shown that transgenic rat lines expressing human truncated tau developed extensive neurofibrillary degeneration, either in the cortex [34] or in the brainstem [35]. The neurofibrillary pathology in the transgenic rat brain fulfils criteria for human NFTs. Importantly, the load of NFTs positively correlated with reactive microglia (Figures 1C and 1D) [34]. Occasionally, activated microglia were closely attached to tangle-bearing neurons (Figure 2), suggesting direct causative interconnection between tangles and reactive microglia [32]. Although there is convincing evidence that diseased, modified tau and neuroinflammation are closely linked, the precise relationship remains unclear.

## Immunosuppression could attenuate tau pathology

The concept based on the premise that anti-inflammatory therapy may prevent or retard the development of AD originated with the discovery that neuroinflammation strongly correlates with AD and that it is associated with senile plaque pathology and NFTs [36,37]. Moreover, epidemiological studies have indicated that nonsteroidal anti-inflammatory drugs (NSAIDs) lower the risk of developing AD [38,39]. In accord with these findings, several drugs, such as the nonselective or COX2-selective NSAIDs and glucocorticoid steroids, were studied to determine if they offer any benefits to AD patients. However, randomized controlled trials assessing COX2 inhibitors (rofecoxib and celecoxib), COX1 and COX2 inhibitors (naproxen and indomethacin) and glucocorticoids (prednisone) failed to demonstrate any beneficial effect on cognition, behaviour or activities of daily living among AD patients [37,40-43]. Furthermore, NSAIDs were not associated with reductions in the load of neuritic plaques (NPs) or NFTs in human AD brain. On the contrary, one study showed that heavy NSAID use was associated with a significant increase in NPs [44]. In contrast, it has been shown that the use of corticosteroids significantly decreased the density of NPs and NFTs in the entorhinal cortices, hippocampi and amygdalae of AD patients [45]. Similarly, immunosuppressive treatment of young transgenic mice expressing mutant tau P301S attenuated tau pathology and increased lifespan. This animal study revealed that targeted inhibition of the inflammatory response can reduce tau pathology and ameliorate disease progression [29].

## Which comes first, neurodegeneration or neuroinflammation?

Whether microglia activation precedes neurodegeneration remains an issue of debate. Several independent studies have shown that neurofibrillary pathology and reactive microglia display the same distribution pattern in AD. The presence of reactive microglia in brain areas affected by neurodegeneration indicates that NFT pathology can drive neuroinflammation. However, some authors have argued that reactive microglia precede tangle formation and thus can establish a template for the development of neurofibrillary degeneration. Sheffield and colleagues [14] showed that microglia occupied the greatest area in periallocortex/allocortex (hippocampus CA1, entorhinal cortex and parasubiculum), a lesser area in association cortex (superior temporal gyrus and orbitofrontal cortex) and the smallest area in primary visual and motor cortex in AD and in controls without AD [14]. Similar findings were described in a mouse model expressing mutated tau P301S, where microglial activation preceded tangle formation [29]. This is only one side of the story; another side points towards age-related structural deterioration of microglial cells that may even precede tangle formation. Wolfgang Streit demonstrated that dystrophic rather than activated microglia correlated with tau lesions [46-48]. Structural and functional impairment of microglial cells may significantly affect the bidirectional communication between tangle-bearing neurons and dystrophic microglial cells. Furthermore, degeneration of microglia would necessarily be associated with a loss of their potential neuroprotective properties. In conclusion, a breakdown of communication between microglia and neurons could contribute to the onset of AD [46-48].

## Cytokine storm can finely tune tau neurodegenerative metamorphosis

Placing cytokine responses upstream in the cascade of deleterious events that lead to tau neurodegeneration has opened new avenues in AD research. Many independent *in vivo *and *ex vivo *studies provide compelling evidence that cytokines can significantly influence the pathological modifications of tau protein. This issue was brought to light by the landmark study of Li *et al. *[49], who showed that reactive microglia producing proinflammatory cytokines such as IL-1 induced an increase in tau phosphorylation in primary cortical neurons. This effect was mediated via the p38 mitogen-activated protein kinase (MAPK) pathways. Similar studies supporting this notion showed that either nitric oxide (NO) or IL-6 can promote tau phosphorylation inside neuronal cells [50,51]. Recently, Lee *et al. *[52] reported that injections of lipopolysaccharide (LPS) into the frontal cortices and hippocampi of transgenic mice rTg4510 expressing human mutant tau P301L induced significant activation of CD45 immunoreactive microglia and increased tau phosphorylation at Ser199/202 and Ser396; however, LPS did not affect the load of silver-positive NFTs. Consistent with these findings, a subsequent study using a 3 × Tg-AD model harbouring PS1_M146V _+ APP_KM670/671NL _+ tau_P301L _mutations revealed increased phosphorylation of tau (p231/235 and p202/205) in the hippocampus [53]. LPS can induce microglial activation and promote hyperphosphorylation of endogenous tau in wild-type mice as well. Increased phospho-tau levels were dependent upon TLR-4 and IL-1 signalling. Moreover, tau phosphorylation was further enhanced in mice lacking the microglia-specific fractalkine receptor (CX3CR1) [54]. Interestingly, gene delivery of the proinflammatory cytokine TNF-α into the brains of 3 × Tg-AD mice induced microglial activation and enhanced intracellular levels of hyperphosphorylated tau [55]. Additionally, a lifetime-based Förster energy resonance transfer (FRET) microscopy study revealed that either activated microglia producing TNF-α or the glia-derived cytokine TNF-α alone were able to induce the accumulation of tau preferentially in neurites [56]. These results strongly suggest that cytokines can significantly modify tau phosphorylation, but have limited impact on tangle formation.

Recent data cast a new light on the role of fractalkine as a key regulator of tangle development. Bhaskar and colleagues [54] showed that transgenic mice lacking CX3CR1 and expressing human tau isoforms had markedly increased numbers of tangle-bearing neurons. This contrasts with a previously published report where the same research group showed that CX3CR1 deficiency led to a reduction of Aβ deposits [57]. It is important to stress, however, that amyloid lesions are located in the extracellular space and that therefore a different mechanism of microglial action could be involved in the degradation of amyloid deposits [57].

On the basis of the data reported so far, it is tempting to hypothesize that molecular dialogue between neuronal cells and microglia can significantly influence neuronal vulnerability to tangle formation. Also on the basis of these data, one could propose that the proinflammatory immune response can amplify tau phosphorylation, which can accelerate tangle formation.

## Genetic background modifies the neuroinflammatory response to tau neurodegeneration

**Figure 3:**
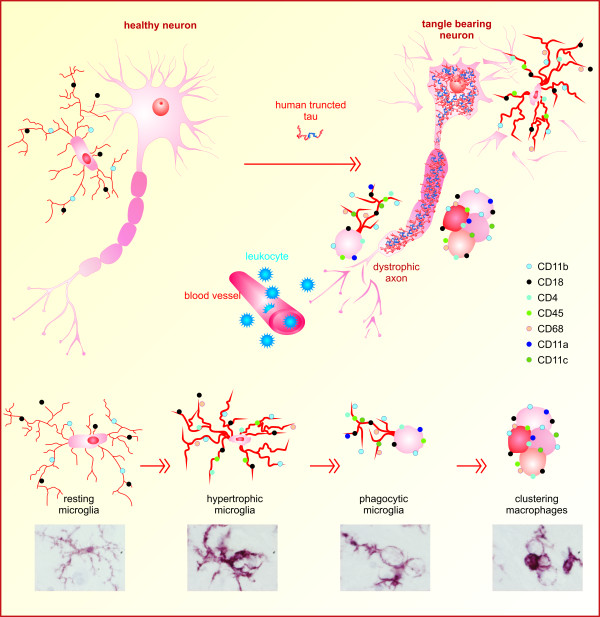
**Functional plasticity of microglial cells in transgenic rat brain expressing human truncated tau protein**. Intraneuronal expression of human truncated tau induces neurofibrillary degeneration. Injured neurons drive resting microglia to become reactive microglia that transform into brain phagocytic microglia: brain macrophages. At this stage, microglia lose contact inhibition and begin to fuse with each other, forming small clusters. Morphological activation of microglia is accompanied with upregulation of several immunological markers, including integrins CD11a, CD11b, CD11c and CD18; lymphocytic antigen CD4; leukocyte common antigen CD45; and lysosomal glycoprotein CD68 (colour dots). In the late stage of neurodegeneration, bloodborne leukocytes (mainly monocytes and partially dendritic cells) infiltrate the brain parenchyma and participate in the brain's immune response.

Numerous epidemiological studies have demonstrated that genetic background modifies the onset and progression of AD and related neurodegenerative disorders. Studies using amyloid mouse models of AD have demonstrated the importance of genetic background for the transgenic phenotype. A significant impact of the genetic background on survival, behaviour, amyloid levels and plaque burden in the brains of amyloid transgenic mice has been observed [58]. To identify genetic modifiers on the tau neurodegenerative and neuroinflammatory cascades, we used transgenic lines expressing human truncated tau protein in either the spontaneously hypertensive rat (SHR) or on the Wistar-Kyoto (WKY) genetic background [33]. The SHR background was chosen for its propensity to develop major risk factors for AD, such as hypertension, insulin resistance and metabolic syndrome. We have shown that tau neurodegeneration can induce inflammatory responses mediated by reactive microglia [32,33]. Activated microglia underwent a transformation from resting to reactive and phagocytic states accompanied by upregulation of several immunologically important molecules, including CD11a, CD11b, CD11c, CD18, CD4, CD45 and CD68. Reactive microglia were frequently distributed around axonal lesions and in close proximity to NFTs. Simultaneously, the innate immune brain response promoted the influx of bloodborne leukocytes (mainly monocytes and partially dendritic cells) into the brain parenchyma (Figure 3) [32]. The WKY rats, which were used as normotensive controls, also developed neurofibrillary degeneration accompanied by neuroinflammation. Moreover, they displayed significantly lower final NFT loads than their SHR transgenic counterparts. We also observed that microglial responses showed a striking difference between transgenic lines. In SHR transgenic rats, a limited number of microglia (less than 2%) expressed major histocompatibility complex class II (MHCII) molecules, despite having a robust phagocytic phenotype. In contrast, almost 20% of the microglia expressed MHCII molecules in WKY transgenic rat brain. Moreover, they displayed a considerably lower extent of phagocytic morphology [33]. These findings suggest that the brain immune response determined by genetic environment could be a potent disease modifier of neurofibrillary degeneration. Whereas tau neurofibrillary degeneration correlates with disease progression, neuroinflammation seems to regulate the resistance or susceptibility to neurodegeneration.

## When the victim becomes the wrongdoer: misfolded tau as a potent activator of the brain's immune response

It has been hypothesized that endogenous intracellular tau may be released into the extracellular space upon neuron degeneration [59]. However, very recent data indicate that tau could be released into the brain interstitial fluid (ISF) in the absence of neurodegeneration. Moreover, the concentration of tau can be significantly higher in the ISF than in the cerebrospinal fluid [60]. These findings strongly point out that intracellular tau is released into the brain's extracellular environment.

Numerous researchers have claimed that soluble extracellular tau may promote neurotoxicity [59,61,62], increase intracellular calcium through neuronal M1 and M3 muscarinic receptors [61], cause synaptic impairment [63] and induce blood-brain barrier damage [64]. We have recently found that misfolded truncated tau is able to activate the innate immune response via MAPK pathways. We reported that purified recombinant soluble truncated tau caused the release of NO, proinflammatory cytokines (IL-1β, IL-6 and TNF-α) and tissue inhibitor of metalloproteinase 1 from mixed astroglia-microglia cultures [65]. It is important to note that the active concentration of misfolded tau was in the range of 0.1 and 1 μM. Similarly, other disease-modified neuronal proteins such as Aβ and α-synuclein are able to activate microglial cells in the same concentration range (Aβ 0.2 to 1 μM and α-synuclein 0.1 to 1 μM) [66-70].

Transcriptomic analysis revealed that truncated tau increased mRNA levels of three MAPKs: c-Jun N-terminal kinase, extracellular-signal-regulated kinase 1 and p38β, which in turn led to the activation of transcription factors such as activator protein 1 and NF-κB. The activation of transcription factors ultimately resulted in enhanced expression of IL-1β, IL-6, TNF-α and NO [65]. These results, together with previously published data, demonstrate that misfolded tau protein is able to induce an innate immune response through a MAPK pathway and that simultaneously tau's pathological transformation can be regulated by activated glial cells via the same pathway.

## Conclusions

**Figure 4:**
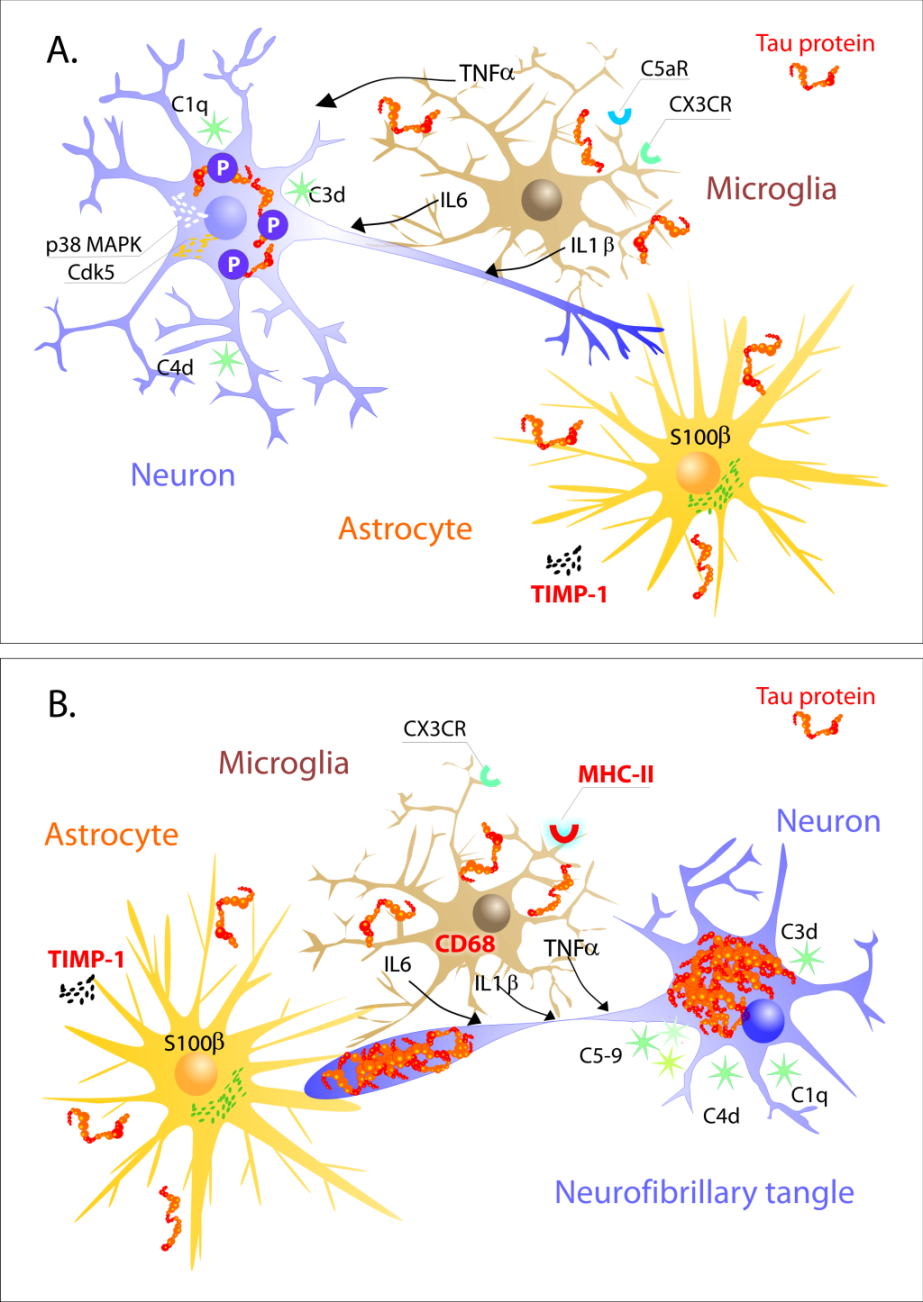
**Tau neuroinflammatory cascade**. In early stages of tau structural metamorphosis, some proinflammatory cytokines (IL-1, IL6 and TNF-α) and chemokines (fractalkine) can modify tau phosphorylation patterns and thus change the structure and function of tau protein. The major sources of proinflammatory cytokines are activated microglia and astrocytes. Glial cells can be activated by different inducers, including misfolded tau protein. At this stage, however, the phosphorylated tau protein would not necessarily aggregate into the filamentous structures **(A)**. In later stages, when misfolded hyperphosphorylated tau proteins form mature NFTs, inflammation could either accelerate or modify tangle formation. Several components of the brain's immune system, including cytokines and proteins of the complement pathway, have been shown to be involved in the molecular dialogue between activated glial cells and tangle-bearing neurons **(B)**.

On the basis of our current body of knowledge, we can conclude that neuroinflammation is a potent modifier of the tau neurodegenerative cascade and, to some extent, can determine the vulnerability of the brain to AD neurodegeneration. The inflammatory response can affect the tau pathological metamorphosis, increasing tau phosphorylation via MAPK kinase pathways and accelerating tangle formation. Extracellular tau can induce immune responses via a MAPK kinase pathway as well (Figure 4). Taken together, these results suggest that tau is located at the crossroad of the neurodegenerative and neuroinflammatory cascades. This is the reason to believe that targeted immunomodulatory therapeutic approaches can significantly modify the tau neurodegenerative cascade and may ultimately have a direct impact on the clinical course of AD.

## Abbreviations

IL: interleukin; NF-κB: nuclear factor κB; TNF-α: tumour necrosis factor α.

## Competing interests

The authors declare that they have no competing interests.

## Authors' contributions

NZ, ZK, SJ, OB, AM and PN each wrote the individual sections in sequential order in the manuscript. DO created all the figures and illustrations. MN coordinated all efforts, made decisions about content and shaped the final form of the manuscript. All authors read and approved the final manuscript.
